# Supplementary material for: Gazing left, gazing right: exploring a spatial bias in social attention
Source: PeerJ. 2023 Jul 12;11:e15694. doi: 10.7717/peerj.15694 (PMC10349552; doi:10.7717/peerj.15694)
Supplement: Supplemental Information 1 — Each code is divided by face orientation, leftwards or rightwards. [file peerj-11-15694-s001.docx]

**Appendix**

KDEF codes of the faces used in the experiment, divided by face orientation:

**Leftwards Rightwards**

AM01NEHL AM01NEHR

AM02NEHL AM02NEHR

AM03NEHL AM03NEHR

AM04NEHL AM04NEHR

AM05NEHL AM05NEHR

AM06NEHL AM06NEHR

AM07NEHL AM07NEHR

AM08NEHL AM08NEHR

AM09NEHL AM09NEHR

AM10NEHL AM10NEHR

AM11NEHL AM11NEHR

AM12NEHL AM12NEHR

AM13NEHL AM13NEHR

AM14NEHL AM14NEHR

AM15NEHL AM15NEHR

AM16NEHL AM16NEHR

AM17NEHL AM17NEHR

AM18NEHL AM18NEHR

AM19NEHL AM19NEHR

AM20NEHL AM20NEHR

AM21NEHL AM21NEHR

AM22NEHL AM22NEHR

AM23NEHL AM23NEHR

AM24NEHL AM24NEHR

AM25NEHL AM25NEHR

AM26NEHL AM26NEHR

AM28NEHL AM28NEHR

AM29NEHL AM29NEHR

AM30NEHL AM30NEHR

AM31NEHL AM31NEHR

AM32NEHL AM32NEHR

AM33NEHL AM33NEHR

AM34NEHL AM34NEHR

AM35NEHL AM35NEHR
